# Utility of Trabecular Bone Score (TBS) in Bone Quality and Fracture Risk Assessment in Patients on Maintenance Dialysis

**DOI:** 10.3389/fmed.2021.782837

**Published:** 2022-01-20

**Authors:** Catalina Poiana, Roxana Dusceac, Dan Alexandru Niculescu

**Affiliations:** ^1^Department of Endocrinology, Carol Davila University of Medicine and Pharmacy, Bucharest, Romania; ^2^Department of Pituitary and Neuroendocrine Disorders, C. I. Parhon National Institute of Endocrinology, Bucharest, Romania

**Keywords:** dialysis (ESRD), trabecular bone score (TBS), fracture risk, dual-energy x ray absorptiometry, bone quality and quantity

## Abstract

Maintenance dialysis is associated with almost universal changes in bone metabolism collectively known as chronic kidney disease—mineral and bone disorder (CKD-MBD). These are accompanied in various proportions by bone loss and altered bone quality that led to an increased risk of fracture. Osteoporosis, age-related or postmenopausal, a condition that often coexists with CKD, is also a leading cause of fracture. Dual-energy X-ray densitometry (DXA) is the main tool for assessing the bone quantity and bone loss and the associated fracture risk. It has been validated in both CKD-MBD and osteoporosis. Trabecular bone score (TBS) is a DXA-derived algorithm for the evaluation of bone microarchitecture, and its clinical value has been repeatedly demonstrated in large cohorts of osteoporotic patients. However, its utility in patients on maintenance dialysis has not been conclusively shown. Published studies showed a lower TBS score and implicitly an altered bone microarchitecture in patients on maintenance dialysis, even after adjusting for various variables. Moreover, FRAX-based fracture risk is higher after adjusting for TBS, showing promise on an algorithm better estimating the clinical fracture risk in dialysis patients. However, TBS has not been demonstrated to independently predict clinical fractures in prospective studies on dialysis patients. Also, aortic calcifications and altered fluid balance could significantly affect TBS score and could hamper the widespread clinical use in patients on maintenance dialysis. In this mini-review, we focus on the benefits and pitfalls of TBS in the management of CKD-MBD and fracture risk assessment in patients on maintenance dialysis.

## Introduction

Maintenance dialysis has been repeatedly associated with an increased risk of hip (1, 2) non-vertebral (2) and vertebral (3) fractures. There are two groups of causes behind this increased risk: those related to the systemic mineral metabolism derangement associated with chronic kidney disease (CKD) collectively known as CKD-mineral and bone disorder (CKD-MBD) (4) and those related to osteoporosis like age, menopausal status, and genetic traits. Interestingly, some factors like age, diabetes mellitus, or medication are related to both CKD and primary osteoporosis. In maintenance dialysis, the factors associated with reduced bone strength are low bone mass, mostly due to secondary hyperparathyroidism and increased bone turnover, and decreased bone quality (abnormal chemical composition and microarchitecture) (5, 6).

Dual-energy X-ray absorptiometry (DXA) is the best tool for predicting fracture risk in the general population (7). Its value has also been proven in maintenance dialysis (8) and was recently added into the guidelines (9). However, its predictive value is significantly lower in dialysis than in the non-CKD population, probably because fracture risk is more dependent on low bone quality in these patients, a feature that is not captured by DXA (6).

Trabecular bone score (TBS) is an index of bone microarchitecture derived from the same lumbar spine DXA scan as the bone mineral density (BMD) (10). Based on the gray-scale variogram of DXA examination, the TBS correlates with the trabecular organization of the cancellous bone independent of the total amount of osseous tissue (11). Ultimately, this correlates with bone resistance and fracture risk (12) in the general population. Moreover, TBS was incorporated in the FRAX tool for assessing fracture risk (13). The major clinical advantages of TBS are low cost and ease of use.

Trabecular bone score (TBS) might be a promising tool for assessing fracture risk in patients on maintenance dialysis. In theory, it might fill the gap between the fracture risk calculated based on hip BMD and the much higher observed risk of hip and non-vertebral fracture. However, the available evidence for the clinical value of TBS in chronic dialysis developed only in the last five years and is far from conclusive. There is only one review of the literature (14) and, since its publication, several important papers became available. The current study aims to review the available evidence on TBS in patients on maintenance dialysis and to provide a basis for future developments in this field.

## Findings of the Available Studies

### TBS in Maintenance Dialysis vs. Controls

The studies on TBS in maintenance dialysis (15–26) are listed in descending chronological order in Table 1.

**Table 1:** Characteristics of the included studies.

**First author**	**Year**	**Study type**	**Controlled**	**Number of subjects**
Yavropoulou et al. (15)	2020	Cross-sectional	Case-control (age, sex, BMI)	30 HD 30 healthy
Yun et al. (16)	2020	Prospective	No	57 HD
Brunerova et al. (19)	2020	Prospective	No	59 HD
Dusceac et al. (20)	2020	Cross-sectional	No	81 HD
Yoon et al. (21)	2019	Cross-sectional	Case-control (age, sex)	76 dialysis (58 HD, 16 peritoneal, 2 transplant) 76 healthy
Dusceac et al. (22)	2018	Cross-sectional	Case-control (age, sex, LS BMD)	98 HD 98 controls
Ramalho et al. (23)	2018	Cross-sectional	No	52 CKD (of whom 33 on dialysis)
Aleksova et al. (27)*	2018	Cross-sectional	No	136 dialysis 10 CKD G5
Aleksova et al. (24)	2018	Cross-sectional	No	137 dialysis 10 CKD G5
Yavropoulou et al. (25)	2017	Cross-sectional	Case-control (age, sex, BMI)	50HD 52 healthy
Perez-Saez et al. (26)	2017	Cross-sectional	Yes	53 dialysis 77 healthy
Luckman et al. (17)	2017	Cross-sectional^#^	No	47 CKD (of whom 23 on dialysis)
Brunerova et al. (18)	2016	Cross-sectional	No	59 HD

* *Same patients as in reference Aleksova et al. (24)*.

# *This is a prospective study but only the baseline (cross-sectional) assessment includes dialysis patients*.

*BMD, bone mineral density; BMI, body mass index; CKD, chronic kidney disease; HD, hemodialysis; LS, lumbar spine*.

Studies that compared TBS in dialysis and control subjects (15, 21, 22, 25, 26) universally found a statistically significant lower score, hence a degraded microarchitecture, in those on dialysis. Most were case-control studies that controlled for age and sex (21), age, sex, and body mass index (BMI) (15, 25), or age, sex, and lumbar spine BMD (22) for a total of 254 cases. Most cases were hemodialysis patients (*n* = 236) while only 18 patients were on peritoneal dialysis and 2 were transplant recipients The largest and smallest TBS differences between dialysis patients and controls were encountered in the study of Yavropoulou et al. (25) (1.11 ± 0.16 vs. 1.3 ± 0.13) and Dusceac et al. (22) (0.07 [0.03–0.1] lower in HD) respectively. Only one study (22) reported the TBS *T*- and *Z*-scores and found them to be is ~0.8 *SD* lower in hemodialysis patients.

As in the general population, TBS was associated with numerous demographics or bone-related factors like age, sex, BMI, or BMD. As a result, accounting for as many as possible of these factors is essential when comparing TBS across different groups. Unfortunately, none of the above-mentioned case-control studies matched non-dialysis patients for more than three factors. However, the homogeneity of the results from different cohorts and methodological approaches suggest that TBS is, indeed, significantly lower in dialysis patients.

Studies that compared TBS with international normative found a TBS lower than 1.31 (28) in 34.2% (21), 42% (23) and 35% (24) of cases. Luckman et al. (17), in 47 CKD patients of whom 23 were on dialysis, found a TBS lower than 1.37 in 53% of cases and Brunerova et al. (18) found a TBS lower than 1.23 in 47.5% of cases. We have to note that the normative used in the above-mentioned studies are derived from the general population and there is no consensus on TBS normal/abnormal values in the end-stage renal disease.

### Predictors of TBS

Most studies found significant correlations between TBS and demographic, bone- or CKD-related factors. As expected, most studies that reported on the association between TBS and age found a significant negative correlation (16–18, 20, 21). Also, most studies found a positive correlation between TBS and BMD (16–18, 20–22, 24), similar to the general population (29). It is interesting to note that TBS correlated not only to lumbar spine BMD (*r* between 0.18 and 0.5) (16–18, 20–22, 24) but also with femoral neck (*r* between 0.25 and 0.4) (17, 18, 20–22) or 1/3 radius BMD (*r* between 0.17 and 0.38) (20, 22, 24). Dialysis vintage inversely correlated with TBS in one study (21) but not in others (22, 24, 26).

As increased bone turnover is present in a significant number of dialysis patients several studies tried to find a correlation between bone turnover markers and TBS. However, the results are heterogeneous with studies reporting inverse (20, 24), direct (16), or no correlations (18, 20, 21, 26). Specifically, Dusceac et al. (20) found a negative correlation between TBS and serum parathyroid hormone (PTH) or C-terminal cross laps of type 1 collage (β-CTx), Aleksova et al. (24) found a negative correlation between TBS and PTH, procollagen type 1-N Propeptide (P1NP) and alkaline phosphatase (ALP) while Yun et al. (16) reported a positive correlation with PTH. Studies reporting an inverse correlation between TBS and bone turnover markers found correlation coefficients around 0.2.

### Mechanisms Behind Low TBS

The most important mechanisms behind low TBS in maintenance dialysis are the same as those in the general population, namely advancing age and low BMD. All studies found robust correlations between TBS and age or BMD and these correlations remained significant in multivariate regression models that included numerous other demographic or clinical factors.

Two interesting studies (17, 23) correlated TBS with structural bone parameters and microarchitecture assessed by high-resolution peripheral computed tomography (HR-pQCT) and histomorphometry from transiliac crest biopsy. Although both studies included also patients with stage 3–4 CKD, most subjects were on permanent dialysis (66 and 48%, respectively) so the conclusions can be extrapolated to this review. Both studies found direct correlations between TBS and parameters of trabecular bone-like trabecular bone volume (BV/TV), trabecular thickness (TbTh), or trabecular width (TbWi), and inverse correlations with trabecular space or trabecular porosity. Moreover, they also found significant associations with the cortical thickness (CtTh), cortical area, or cortical density. Unexpectedly, HR-pQCT findings of increased cortical bone with higher TBS were paralleled by a lower cortical width at histomorphometry. It is worth mentioning that all these findings were heterogeneous between radius HR-pQCT, tibia HR-pQCT, and iliac crest histomorphometry. As expected, both studies reported significant correlations with areal or volumetric BMD.

In a recent study, Yavropoulou et al. (15) evaluated the serum concentrations of bone metabolism-related microRNAs in 30 patients on permanent hemodialysis and found that some of these small molecules were significantly downregulated compared to healthy controls. MicroRNA-23a-3p, which targets the runt-related transcription factor, was specifically inversely correlated with TBS (rho = −0.503) and remained significant after adjusting for BMD.

Also, in a recent study by Yun et al. (16), TBS was prospectively inversely associated with new-onset cardiovascular events (coronary artery disease, stroke, or peripheral arterial occlusive disease), suggesting that profoundly altered mineral metabolism leads to deranged microarchitecture and vascular calcifications.

Further findings showed that three studies (16, 20, 24) tried to find a correlation between the etiology of CKD and TBS or BMD. Aleksova et al. (24) found significantly lower TBS and LS or femoral neck BMD in those undergoing simultaneous pancreatic kidney transplantation (those with type 1 diabetes mellitus). Similar to the general population, Dusceac et al. (20) found a significantly higher LS BMD in patients with diabetic kidney disease vs. other etiologies but no differences in TBS. Lastly, Yun et al. (16) found no difference in TBS in dialysis patients with or without diabetes.

### Fracture Risk

The majority of studies also reported data on fracture and fracture risk (16, 19–21, 23–26). Most of the data come from cross-sectional studies that compared TBS between patients with and without prevalent fractures (21, 23–26). Four of these studies reported similar TBS between dialysis patients with and without fracture (21, 23, 25, 26) while Aleksova et al. (24) found significantly lower TBS in patients with non-vertebral fractures. Although four out of five studies reported negative results (non-significant differences), the TBS was lower, albeit non-significant in patients with prevalent fracture suggesting that lack of statistically significant results might be due to underpowering. Also, in a cross-sectional study, Dusceac et al. (20) found that the risk of fracture calculated by FRAX increased after adjusting for TBS in hemodialysis patients and was significantly higher than in age, sex and BMD matched controls.

Two prospective studies measured TBS at baseline and followed the patients for 20 (16) or 24 (19) months. The results were heterogeneous, with one study (16) showing lower TBS in those with incident fractures (1 vertebral, 1 hip, 2 lower extremities, and 3 upper extremities) while the other (19) found no significant differences (1 vertebral, 2 hip, 2 forearms, 1 humerus, and 1 rib).

### Pitfalls of TBS

The trabecular bone score measures trabecular bone and microarchitecture at the lumbar spine level and was not developed for other skeletal sites. However, hip fracture is the most prevalent type of fracture in maintenance dialysis with rates 1.7 to 98 times higher than in the general population, depending on the age and sex (1). There are concerns that TBS, captured at the lumbar spine level, might not reflect hip microarchitecture and hence, hip fracture risk. Currently, there is no study in maintenance dialysis associating low TBS with a higher incidence or prevalence of hip fracture. However, available studies found robust correlations between lumbar spine measured TBS and hip (17, 18, 20–22) or 1/3 radius (20, 22, 24) measured areal BMD. Also, studies found significant associations between TBS and various parameters of trabecular microarchitecture measured by HR-pQCT at the ultradistal radius or tibia or by histomorphometry at the iliac crest. Taken together, these findings suggest that lumbar spine measured TBS has the ability to capture the widespread skeletal alterations characteristic of CKD-MBD. Moreover, in the general population, TBS was shown to predict hip fracture independently of BMD and age (12).

The trabecular bone score is measured at the lumbar spine level, a region commonly affected by osteoarthritis and, more importantly, by vascular calcification so prevalent in maintenance dialysis. These led to speculations that TBS might not reflect the actual bone microarchitecture in chronic dialysis. Available data show that, at least in the general population, TBS is not affected by spinal osteoarthritis (30). The only study that tackled the relation between aortic calcifications and TBS in patients on permanent dialysis (27) showed an inverse correlation between these parameters, a finding interpreted as TBS and bone microarchitecture are an expression of the deranged mineral metabolism of CKD. In the same study lumbar (27) spine BMD was not associated with aortic calcifications. It is worth noting that in the study of Dusceac et al. (20) the correlations lines between TBS and lumbar spine BMD in patients and BMD-matched controls run parallel, suggesting a lack of significant derangements of aortic calcifications on TBS.

Also, there are some other putative factors like image noise, BMI, or water content of soft tissue that could limit TBS use. Image noise tends to lower TBS but the clinical impact is very low (31). Similarly, although BMI or fluid retention (in relation to dialysis timing) might impact TBS, the clinical relevance is minimal (32, 33). Moreover, the vast majority of dialysis patients included in the cited studies did not have extremely low or high BMI.

## Discussion

The trabecular bone score was shown to reflect bone microarchitecture in the general population. Its value in fracture prediction, beyond that of classic clinical factors and BMD, was consistently demonstrated (12) and TBS was added to the FRAX tool (13). Maintenance dialysis is associated with profound derangements of the mineral and bone metabolism that ultimately lead to an increased risk of fracture (34). Unfortunately, the power of fracture risk prediction in maintenance dialysis is far below that of the general population (34). This review gathers the available evidence on the utility of TBS in predicting bone status and fracture risk in maintenance dialysis.

The magnitude and the consistency of the available data, combined with the diversity of the approaches, confirm that TBS is a valuable tool in the evaluation of the mineral and bone status of chronic dialysis. TBS was found to be lower in maintenance dialysis compared with healthy subjects (15, 21, 22, 25, 26), a feature that was somehow expected given the high fracture risk of CKD. Moreover, these findings are in concordance with those from patients with stage 3–4 CKD (35). As the TBS is lower independently of age, sex, BMI, or BMD, it must capture features of bone and mineral metabolism other than those predicted by these parameters. On the other side, TBS is significantly correlated with all these parameters, so it is tempting to suggest that TBS reflects altered microarchitecture due to both classic factors and those related to maintenance dialysis. HR-pQCT and histomorphometry studies confirmed that the trabecular score is based on actual trabecular bone parameters: trabecular thickness, trabecular space, or bone volume (17, 23). Studies correlating TBS with vascular calcifications (27) and cardiovascular events (16) proved that TBS is more than a “bony” parameter.

The pathogenesis of low TBS and altered microarchitecture in maintenance dialysis is far from clear. The available evidence suggests that it is not related to increased bone turnover because TBS correlation with bone turnover markers is not significantly anymore after adjusting for BMD (20). Unfortunately, the study that associated TBS with osteoblasts and osteoclastogenesis *via* microRNAs did not adjust for BMD (15). This is very important from both pathogenic and therapeutic points of view as treatment with antiresorptive, drugs that dampen bone turnover, might not have the same effects as in the general population. Interestingly, this stands against the value of denosumab in dialysis patients (34).

The etiology of CKD can have a direct effect on BMD and TBS. For example, it is known that patients with type 1 diabetes mellitus (DM) have decreased while those with type 2 DM have increased BMD compared with controls (36) while TBS is significantly lower (37). Results on TBS in dialysis patients were heterogeneous, with studies finding both significant (24) and non-significant (16, 20) differences between patients with or without diabetes. These might be due both to the type of DM assessed in the study (T1DM or/and T2DM) and the number of subjects, which was significantly lower than in studies on diabetic patients with normal renal function. Moreover, the “control, non-diabetic” group of dialysis studies is composed of patients with various pathologies that might affect bone structure and TBS. Autosomal dominant polycystic kidney disease (ADPKD) has been proposed to have a distinct bone phenotype with preserved cortical BMD due to suppressed bone turnover (38). The only study that quantified TBS in ADPKD could not find a significantly different TBS vs. other CKD etiologies (20).

The trabecular bone score shows a promising value in fracture prediction. It demonstrated a trend toward lower values in those with prevalent or incident fractures (21, 23–26). Moreover, correction of classical FRAX for TBS increased the risk of fracture (20). The usefulness of TBS in fracture risk prediction could be double. In older patients, in whom FRAX is better validated, TBS could provide the tuning of the risk with observed values. However, TBS could be significantly more important in young patients on dialysis in whom both BMD and FRAX are poorly validated (34). We also have to keep in mind that FRAX also adjusts for mortality and the life expectancy of chronic dialysis is greatly reduced. It is important to note the relative risk of fracture (vs. age-matched general population) is significantly higher in young than in older patients (1).

There are significant drawbacks in the available studies. Studies design is probably the most important, as the controlled studies are cross-sectional while the prospective ones are not controlled and not adequately powered. Moreover, many studies do not have hard endpoints like radiological or clinical fractures. Also, the number of patients on peritoneal dialysis is underrepresented.

In conclusion, TBS shows promising value in maintenance dialysis for both bone and vascular endpoints. Particularly, fracture risk prediction might benefit most from routine TBS assessment. Future studies should focus on prospectively, adequately powered studies with clinical and radiological fracture endpoints.

## Author Contributions

CP, RD, and DN collected and analyzed the data, wrote, reviewed, and edited the manuscript. All authors contributed to the article and approved the submitted version.

## Funding

This research received funds for open access publication fees from Carol Davila University of Medicine and Pharmacy.

## Conflict of Interest

The authors declare that the research was conducted in the absence of any commercial or financial relationships that could be construed as a potential conflict of interest.

## Publisher's Note

All claims expressed in this article are solely those of the authors and do not necessarily represent those of their affiliated organizations, or those of the publisher, the editors and the reviewers. Any product that may be evaluated in this article, or claim that may be made by its manufacturer, is not guaranteed or endorsed by the publisher.
